# A scientometrics study of the nanomedicines assisted in respiratory diseases

**DOI:** 10.3389/fbioe.2022.1053653

**Published:** 2022-12-02

**Authors:** Yi Yang, Dexu Luo, Muhammad Inam, Jialin Hu, You Zhou, Chuanshan Xu, Wenjie Chen

**Affiliations:** ^1^ Guangzhou Municipal and Guangdong Provincial Key Laboratory of Molecular Target & Clinical Pharmacology, The NMPA and State Key Laboratory of Respiratory Disease, School of Pharmaceutical Sciences and the Fifth Affiliated Hospital, Guangzhou Medical University, Guangzhou, China; ^2^ Guangdong-Hongkong-Macao Joint Laboratory of Respiratory Infectious Disease, Guangzhou, China; ^3^ Sydney Vital Translational Cancer Research Centre, St Leonards, NSW, Australia

**Keywords:** nanomedicine, respiratory disease, scientometrics analysis, COVID-19, mRNA vaccine

## Abstract

Nanomedicine has been extensively studied for its versatility and broad-spectrum applications of theranostics in the research of respiratory disease. However, to the best of our knowledge, a scientometrics study based on the scientific knowledge assay of the overall situation on nanomedicine applied in the research of respiratory disease has not been reported so far, which would be of major importance to relevant researchers. To explore and exhibit the research status and developing trend of nanomedicines deployed in basic or clinical research in respiratory disease, the research ecosystem and exciting subareas were profiled based on the massive data mining and visualization from the relevant works reported from 2006 to 2021. Data were collected from the Web of Science database. Data statistics software and bibliometric analysis software were employed to visualize the research trend and the relationship between respiratory diseases and nanomedicines in each representative direction. The cluster analysis and burst detections indicated that the improvement of drug delivery and vaccine developments are the up-to-date key directions in nanomedicines for respiratory disease research and treatments. Furthermore, we emphatically studied four branch areas in this field including COVID-19, nanotube, respiratory syncytial virus, and mRNA vaccine those are selected for in-depth mining and bibliometric coupling analysis. Research trends signify the future focuses will center on preventing respiratory diseases with mRNA vaccines using nanoparticle-based approaches. We anticipate our study will enable researchers to have the panorama and deep insights in this area, thus inspiriting further exploitations especially the nanobiomaterial-based systems for theranostic applications in respiratory disease treatment.

## 1 Introduction

Air pollution, climate change and microbial infections are the main factors causing respiratory diseases (Almetwally et al., 2020). Among the common diseases of high incidence, the respiratory diseases inflict patients suffering from lung and bronchial lesions, with the symptoms including making cough, expectoration, breathing difficulties, asthma, and respiratory disorders, which will gradually affect lung function and lead to the lung dysfunction, respiratory failure and even death (Moelling and Broecker, 2020). For most drugs treating respiratory diseases, inhaler devices and strict formulations are required using high technical requirements, so an smart and effective design is highly demanded for respiratory drug delivery (Newman, 2018).

Nanoparticles (NPs) are artificially or biomimetically manufactured particles that can penetrate cell membrane and spread along nerve cell synapses, blood vessels, and lymphatic vessels (Mitragotri et al., 2015) so that they are widely applied in disease diagnosis, treatments, and imaging (Singh and Lillard, 2009). Nano drug delivery systems can be armed with capabilities of localized targeting and controlled drug release, which provides a revolutionized way to diagnose and treat respiratory diseases (Pison et al., 2006). The commonly used nanomedicines mainly include liposomes (Komalla and Haghi, 2020), solid lipid nanocarriers (Nassimi et al., 2010), polymer nanocarriers (Smola et al., 2008), dendritic polymers (Stiriba et al., 2002), inorganic nanocarriers (Liang et al., 2014), and protein nanocarriers (Elzoghby et al., 2012), which served as the versatile platforms for different therapeutics in fighting against respiratory diseases. For example, chemotherapy drugs such as doxorubicin (DOX) or paclitaxel (PTX) can be delivered through liposomes to treat lung cancer (Landesman-Milo et al., 2015). In addition, for treating the chronic obstructive pulmonary disease, nanosystem-based drugs such as amikacin (AM) and gene therapy have been shown remarkable enhancement in cell targeting and therapeutic effectiveness (Li et al., 2020). This area has attracted intensive attentions and several anti-lung cancer nanomedicines have already been approved clinically (Norouzi and Hardy, 2021). For fighting against the tuberculosis (TB), which is the second leading infectious killer only after COVID-19, nanomedicines such as the cross-linked poly-β-cyclodextrin (pβCD) has been applied as an efficient carrier for delivering anti-TB drugs to the lung, which promoted the apoptosis of macrophages and thus preventing the infection process (Machelart et al., 2019). Apart from the anti-TB applications, NPs can also be employed in the early diagnosis of TB, a dot-blot immunoassay using the gold-copper nanoshell was reported to monitor the early onset of tuberculosis by detecting 10-kDa culture filtrate protein (CFP-10) (Welin et al., 2015). The current unending COVID-19 pandemic which is caused by the severe acute respiratory syndrome-coronavirus-2 (SARS-CoV-2) virus has led to a huge number of infections and deaths in recent 2 years. The virus is highly transmittable but presently incurable with no specific remedies, which makes the preventive vaccines such vital to contain the virus. In the developments of anti-SARS-CoV-2 vaccines, NPs have performed their dispensable roles. mRNA-lipid nanoparticles (LNPs) have recently been propelled onto the center stage of therapeutic platforms due to the success of the SARS-CoV-2 mRNA LNP vaccines (mRNA-1273 and BNT162b2) (Kon et al., 2022). LNPs provide a promising direction for developing the SARS-CoV-2 vaccines and other vaccines for the incurable virus-infected diseases (Moitra et al., 2020). Similarly, another novel NPs-based vaccine has been also applied for combating against another deadly respiratory virus, middle east respiratory syndrome coronavirus (MERS-CoV), in this formulation, the delivery of interferon (IFN) gene agonists and irritant subunits of viral antigens were based on the poly (lactic-co-glycolic acid) (PLGA) hollow nanocarrier (Lin et al., 2019).

In the past 2 years, increasing efforts have contributed to the studies of nano theranostics for the unmet needs of fighting against the epidemic of SARS-CoV-2. Some reviews published previously mainly discussed various applications of nanoparticles applied in respiratory diseases or disorders, the research status of nanocarriers based on the physicochemical properties (Luo et al., 2021), as well as the advantages and disadvantages of applying nanotechnology (Omlor et al., 2015; de Menezes et al., 2021; Zhong et al., 2021). However, these reviews have not done an analysis of the state of the research at different times and the popularity of different themes in a visualized and panoramic manner. To our knowledge, there has no bibliometric analysis of nanomedicine used in respiratory diseases been reported so far. To this aim, this study mainly focuses on the visualized analysis of multiple fields crossing between the nanomedicine and respiratory diseases, we quantitatively analyzed and map all the published literatures, explored the whole developing hotspot, and foresee the evolutional and future development trend.

## 2 Data and methods

### 2.1 Data acquisition

Web of Science Core Collection (WoSCC) is one of the largest and most comprehensive academic databases covering the most disciplines, including the most influential core scholarly journals in natural science, engineering technology, biomedicine, and other research fields (Wáng et al., 2014). In addition to searching literature, it can also provide important information on citation index. Researchers can use the retrieval function of the citation index, catch up with the research status of a particular field and obtain relevant information from global academic community through WOS. In this study, respiratory diseases and nanoparticles are our primary research subjects, therefore the retrieval query was set as “TS = (respiratory disease* OR respiratory disorder*) AND TS = (nanoparticle* OR nanocarrier* OR nanomedicine*)” for data search and analysis. The search of the core collection on topics related to respiratory diseases and nanoparticles from 2006 to 2021 hit 1,125 records of published articles. Data were exported with the text files formatting with full details including the author, institution, country, abstract, keyword, date, cited reference, etc.

### 2.2 Method

Microsoft EXCEL 2016 was used for statistical analysis and graph plotting. Bibliometric analysis and data visualization were carried out using two bibliometric tools, CiteSpace (Chen, 2006) and VOSviewer (van Eck and Waltman, 2010). In this study, CiteSpace was used to 1) overlap the country and author’s dual map; 2) analyze the publishing keywords; 3) analyze the co-citation; 4) analyze the references for several prominent keywords; and 5) analyze and quote the top-cited references. In the keyword analysis, the centrality was determined according to the frequency of simultaneous citation in the coupling analysis of cited literature with different subject words.

The bibliographic coupling assay is carried out in the software, CiteSpace, which gives indicators to evaluate the clustering. The indicator used to evaluate the significance is the silhouette coefficient ( $S\left(i\right)$ in the Eqs. 1, 2) (Goh et al., 2002), and the centrality indicator for the nodes of the co-citing network is betweenness centrality or centrality (*ρ*_*jk*_ in the Eq. 3) (Newman, 2005). These two indicators are defined in the following Eqs 1–3

$$S\left(i\right)=\left\{\begin{array}{c} 1-a\left(i\right)/b\left(i\right),\;ifa\left(i\right)<b\left(i\right),\;\\ 0,\;ifa\left(i\right)=b\left(i\right),\\ b\left(i\right)/a\left(i\right)-1,\;if\;a\left(i\right)>b\left(i\right) \end{array}\right.$$ (1)

$$-1\leq s\left(i\right)\leq 1$$ (2)

$$Centrality\left({node}_{i}\right)=\underset{i\neq j\neq k}{\sum }\frac{p_{jk}\left(i\right)}{p_{jk}}$$ (3)

In Eq. 1, for each datum i in dataset, $a\left(i\right)$ is the average distance of i from all other data within the same cluster, $b\left(i\right)$ is the lowest average distance of i from any other cluster of which i is not a member, and $S\left(i\right)$ is the silhouette coefficient and falls in the range of [−1, 1], as shown in Eq. 2. In Eq. 3, $p_{jk}$ represents the number of shortest paths between node j and node k , and $p_{jk}\left(i\right)$ is the number of those paths passing through the node i . At the document level, the importance of each document in a co-citing network can be partially evaluated by the indicator centrality.

## 3 Results and discussion

### 3.1 Annual publications and citations

Basically, the publications are wildly recognized as the referable index for showcasing a specific research field. To understand the developments and research trends of nanomedicines used in the respiratory disease in recent two decades from a macro perspective, the timespan ranging from 2006 to 2021 was set to retrieve data from the database WoSCC for the following assays.

**FIGURE 1:**
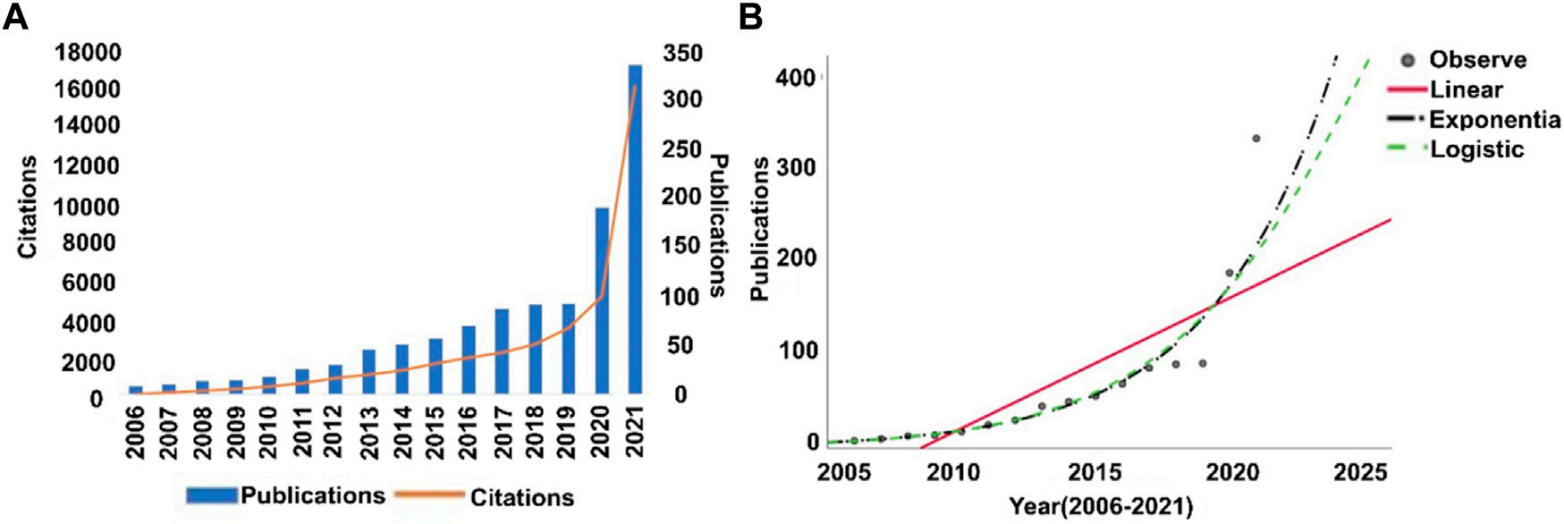
Analysis of annual publications and citations. **(A)** Annual distribution of publications and citations in the area of nanoparticles used in respiratory diseases. **(B)** The fitting curves of publications on nanomedicines used in respiratory diseases in WOS (2006–2015).

Annual publications and citations can directly reflect the historical trend of a specific research field. After a series of literature screenings, 1,125 published items were retrieved. Publication distribution and citation frequency are shown in Figure 1A. As we can see, in the past 15 years, despite no significant growth from 2017 to 2019, the annual publications in this area showed an ascending tendency. Noteworthily, the number of articles published in 2020 showed a sharp increase, with the number of publications doubling that of last year, it maintained this trend of rapid growth in 2021. The citations also exhibited the same tendency as the publication distribution. Cumulative total citations were 41,627 times, with 39,901 times after removing self-citations, reaching an average of 36.77 citing times per publication. Furthermore, a non-linear simulation of the inclination of publications was conducted, as shown in Figure 1B, it reveals that the growth pattern in Figure 1A appears closer to the logistic or exponential function, with R^2^ values larger than 0.95, which indicates good fittings of the non-linear moldings. The statistical indicators in Figure 1B are shown in the Supplementary Table S1, the exponential regression (R^2^ = 0.972) appears to be the best fitting curve, to which the logistic fitting (R^2^ = 0.959) is very close.

It should be noted that in the last 2 years, publication numbers in 2020 and 2021 almost doubled compared to the last year, respectively, which reflects the sharply growing attentions in this area in recent 2 years of COVID-19 pandemic have contributed to large amount of research output.

### 3.2 Analysis of countries, regions and institutions

A geographical distribution map depicting each country’s contribution is visualized as shown in Supplementary Figure S1A with 20 countries counted. According to the color brightness, it can be observed that most of the studies are mainly from the United States, People’s Republic of China, India and Australia etc. Most research papers have been published in Europe and the United States, where research on respiratory diseases and nanoparticles is most active. Specifically, as shown in Supplementary Table S2, the United States published the most papers with the highest citation in this field. India has the second-highest number of articles published. Predictably, the number of documents in People’s Republic of China may increase shortly.

Collaboration is an important way to enhance research quality and productivity. As shown in Supplementary Figure S1B, the United States has the most links with other countries, followed by China, Germany etc. In addition, according to the density of crossed lines, research links between European countries are much closer than other areas. Asian countries have fewer communications with other countries except for People’s Republic of China and India. It is anticipated that more Asian countries will be seen participate in this field in the short run.

To analyze each country’s contribution over time from 2006, as shown in Supplementary Figure S1C. The United States has been involved in this area since 2006, which is the earliest, and continued to contribute in the following years. After the year of 2006, especially from 2007 to 2011, a large number of other countries started to participate in this area. Since 2015, Asian countries such as Malaysia began to research in this area.

To analyze the institutions that contributed to the field, Supplementary Figure S2A sketched *via* Citespace has shown the most prolific institutions including University Technology Sydney, Chinese Academic of Science, University of Maryland, which are conspicuous with large font, indicating that they have made major contributions to this area. The influence can be indicated through their number of publications, which also have drawn high citations.

Institutions, their affiliated countries, and their research directions are shown in Supplementary Figure S2B, show countries with a representative institute and the major direction, the majority of research were produced from academic institutions and only a few biopharma companies such as BioNTech.

### 3.3 An overview of research hotspots and frontiers

#### 3.3.1 Analysis of keywords

**FIGURE 2:**
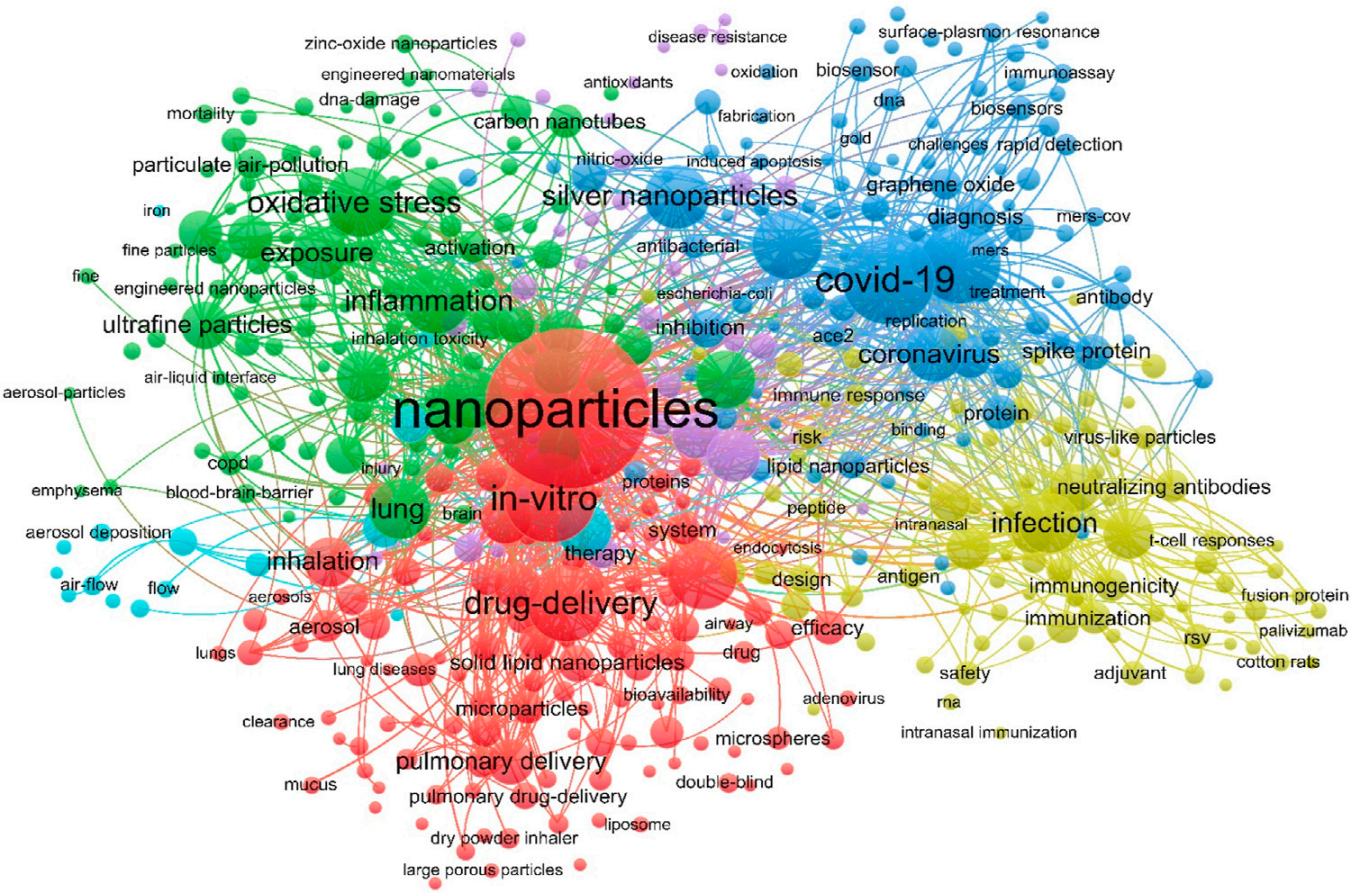
The network map of the keyword clusters in the area of nanoparticles used in respiratory diseases.

Keywords represent the main themes and core substance of a specific publication, which also enable us to legibly recognize hot research themes in the co-occurrence analysis of a specific area. By counting the minimum frequency occurrence more than 5 times, 447 keywords were selected for the quantitative analysis. Figure 2 represented the overlay visualization map of the most frequently used keywords in this area. The size of each node shows the keyword centrality, that means large nodes with more branches have high centrality of this keyword.

There are six clusters with six colors. The red colored cluster 1 contains 117 keywords, such as “drug delivery” and “*in vitro*”. Generally, nanoparticles are used in different delivery methods, especially for the purpose to target organs or cells (Wilczewska et al., 2012), thus “delivery” is a very important keyword. The green colored cluster 2 consists of 113 projects related to nanoparticles, “inflammation” and “oxidative stress”, which are key keywords significantly affecting the normal function of the respiratory system. The blue colored cluster 3 has 102 keywords, mainly focus on the delivery of nanoparticles, “COVID-19” is the largest node and the hottest keyword. COVID-19 and coronavirus those are newly emerging hotspots, because of the current COVID-19 epidemic, which may continue to be intensively studied. Clusters 4, 5, and 6 are the smaller clusters with 85, 39, and 16 keywords, respectively, which particularly display the research aspects in nanovaccine against infections (yellow cluster), nanotoxicity and disease resistance (purple cluster), and inhalation approaches (cyan cluster), respectively.

#### 3.3.2 Analysis of co-citation references

**FIGURE 3:**
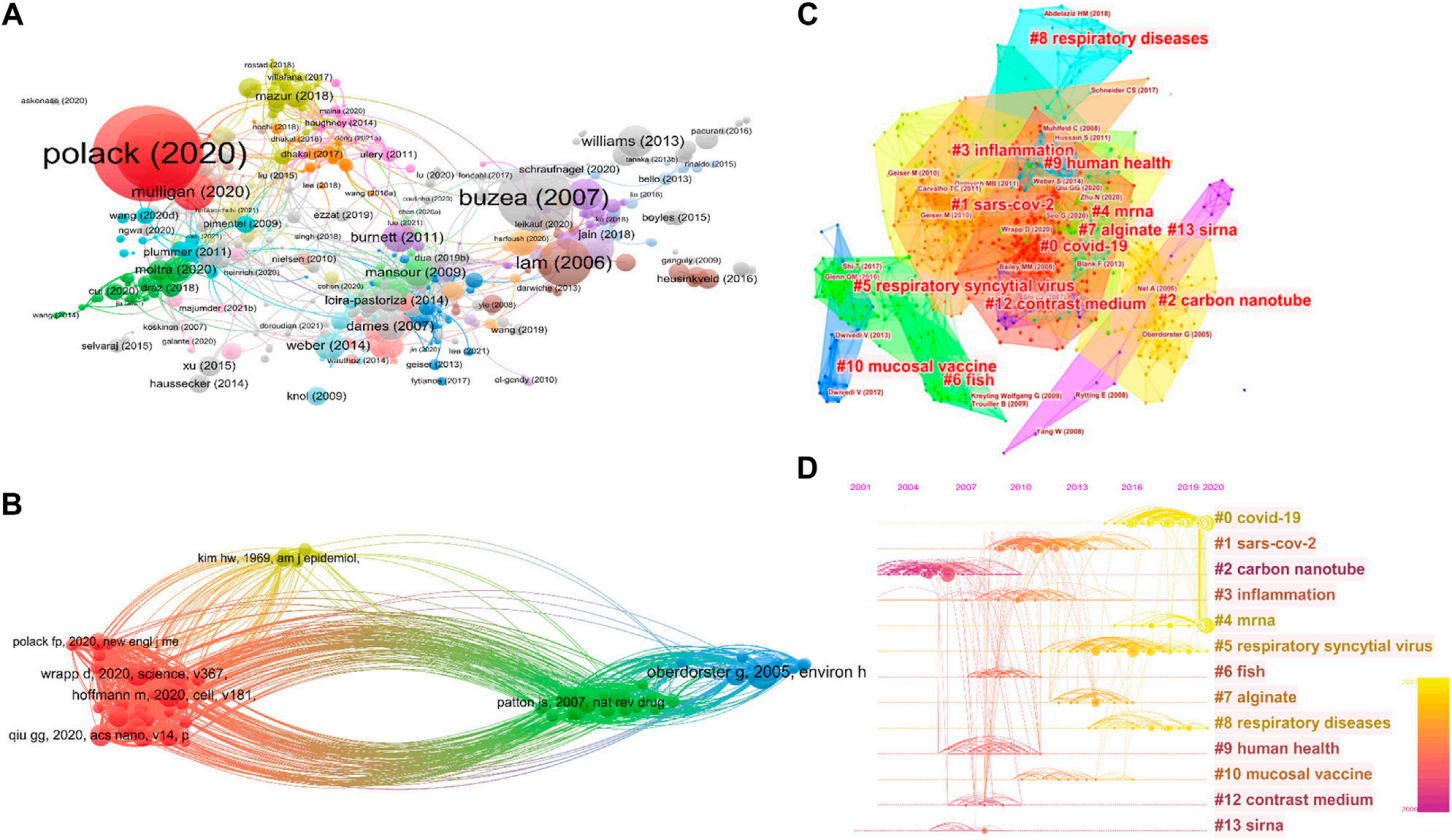
Analysis of the co-cited reference. **(A)** Visualization graph of cited references. **(B)** The connection between references. **(C)** Co-citation references keywords. **(D)** The timeline of cited references.

The co-citation analysis of references can evaluate the evolution and trace the developmental frontiers of any subjects. The data was imported into VOSviewer. A visualization graph of cited references was plotted shown in Figure 3A, there are 1,125 documents forming the corresponding nodes. The results indicated for the highest cited document, Polack verified the safety and efficacy of the COVID-19 vaccine produced by BioNTech and Pfizer (Polack et al., 2020). The third most cited document is also about the safety and efficacy of a COVID-19 vaccine developed by the company Moderna (Baden et al., 2020). These two companies developed COVID-19 vaccines, both using the LNP-formulated mRNA vaccines expressing the SARS-CoV-2 antigen domain. It can be inferred that since the outbreak of COVID-19, researchers have shifted enormous interests to the safety and effectiveness of COVID-19 vaccines.

The document shown by the second-largest node is about nanoparticles’ origin, activity and biological toxicity (Buzea et al., 2007). Another review on the toxicity of CNTs also has a large number of citations (Lam et al., 2006). The toxicity of nanoparticles has been a hot topic of research for a long term. In specific, the particulate matter (PM) and nanoparticles (NPs) can generate oxidative stress and thus causing the oxidant injury (Li et al., 2008), which has been extensively studied in the area of nanotoxicology.

In Figure 3B, influential and representative nodes with many emanative arcs linked 4 clusters and 64 items. The first clusters mainly comprise the articles published in recent 2 years concerning on how the SARS-CoV-2 virus invades the human body and how to treat the infection (Hoffmann et al., 2020). COVID-19 has been the core research hotspot that drew more and more efforts. For example, inhaled drugs are designed to be administrated through the lungs, which is principal for the delivery systems of drugs targeting the lung tissue (Patton and Byron, 2007). For the study of pulmonary delivery mechanisms, one exemplified article (Patton, 1996) demonstrated another hotspot of pulmonary drug delivery using NPs.

Furthermore, a citation relationship is formed when two or more references are cited in the same document. In Figure 3C, citation analysis clusters displayed clustering keywords and literature citation network, providing 3,230 links among them, 13 clusters in total, and the centrality values of each node were listed in Supplementary Table S3. Modularity value (Q-value) and mean silhouette value (S-value) are two significant elements to evaluate the community structure, Q > 0.3 and S > 0.7 are denoted to a significant clustering (Li et al, 2021). In this study, the Q-value was 0.8403, indicating the rationality of this network. The mean S-value was 0.9452, suggesting the good dispersity of these clusters. Figure 3C visualized the keywords including “COVID-19,” “carbon nanotube,” “inflammation,” “mRNA,” “RSV,” “fish,” “alginate,” “respiratory diseases,” “human health,” “mucosal vaccine,” “contrast medium,” and “siRNA,” some of which are nanomedicines used in the research of respiratory disease diagnosis, prevention and treatment. Mucus clearance is the basic defense to protect the respiratory system from inhaling infectious or toxic substances (Button et al., 2012). Through the citation analysis, three papers about COIVD-19 have been cited mostly, showing that the research on COVID-19 is mostly based on fundamental medical research. A review paper with the highest centrality on the nanoparticle-based vaccines introduced some novel preparation methods using various nanoparticles such as virus-like particles and protein-like particles to deliver antigens, which differs from the traditional vaccine and showed promising efficacy in preventing respiratory diseases (Al-Halifa et al., 2019). Another study elucidated how the RSV virus causes respiratory tract infections those are probably related to the seasonal change (Garlapati et al., 2012), which has a very high centrality of 0.35 among those references.

To investigate the evolutionary trend of the main topics and their interactions, the timeline of the references has also been mapped. As shown in Figure 3D, the inflammation has always been a focal topic in the biomedical area due to its universal existence in many physiopathologic circumstances. Considering that nanoparticles can also enter the healthy tissues through the respiratory tract, causing unhealthy reactions including the inflammation, hence the inflammation has undoubtedly become the research focus in the progress of inhalation-induced diseases. Especially with the changes in the ecosystems and environment, including air pollution, particularly the toxic compounds carried by PM, and other factors including the unhealthy habits like smoking, second-hand smoke, those have been demonstrated to be significantly associated with the occurrence of respiratory inflammation and even lung cancers (Arias-Pérez et al., 2020). The air quality seems to get deteriorate if without effective interventive measures taken, and the intensive studies on the hazardous airborne NPs with pathogenicity and or favorable artificial NPs for therapeutic research, will continue to grow with considerable speed in the future time, which will benefit us to research and treat the respiratory inflammation and other diseases. While in the specific era of COVID-19, the mRNA, LNPs as well as respiratory syncytial virus became the hotspots simultaneously. The research on those topics will last for an extended period since the epidemic remains unfinalized. It is prospective that in the area of drug delivery or diagnosis of respiratory disease, nanoparticles will keep being of great importance to developing effectual treatments.

#### 3.3.3 Analysis of the citation bursts

To capture the rapid increases of popularity of the references or keywords within a period, the top 25 cited literature were analyzed, and the results were shown in Supplementary Figure S3. The dark green dashes in the chart represent the time when a paper published and the red dashes represent the year when the paper was cited prevailingly, while the light ones show the years the paper have not been published yet. The rapid development of nanotechnology increased the awareness to study risk of respiratory diseases, so the subject of nanotoxicology is found bursting, which concentrates on the evaluation of the safety and toxicity of engineered nanostructures (Oberdörster et al., 2005). Hence, from 2006 to 2013, the highly cited articles investigated the toxicity and safety of nanoparticles, researchers in this period primarily focused on the toxicity of nanoparticles. An article (August et al., 2017) on nanotoxicology has been cited frequently for a long time, in briefly, this article conceptually introduced nano-toxicology and how nano-toxicology is derived, providing many fundamental viewpoints with vital significance to guide the follow-up research. At present, several nanoparticle-based vaccines have been in the stage of clinical research, and mRNA vaccines show a rapid growing trend from 2018 to 2021 (August et al., 2017). With the development of nanovaccine technology, the demand for nanoparticle-based vaccine studies in clinical would be increasingly growing. For instance, a study published in 2016 reported the nanovaccine for women at childbearing age against respiratory syncytial virus (RSV) that under clinical research, it was highly cited as soon as it was published, demonstrating that this paper provided a rational design for most of the studies in clinical trials of RSV nanovaccine. In particular, it will inspire the following research and more importantly the clinical trials of RSV nanoparticle-based vaccine (Glenn et al., 2016). Implicating by the trend of citations, there will be more clinical studies of nanoparticle-based vaccines for respiratory diseases in the future.

#### 3.3.4 Analysis of representative subareas *via* bibliographic coupling analysis

**FIGURE 4:**
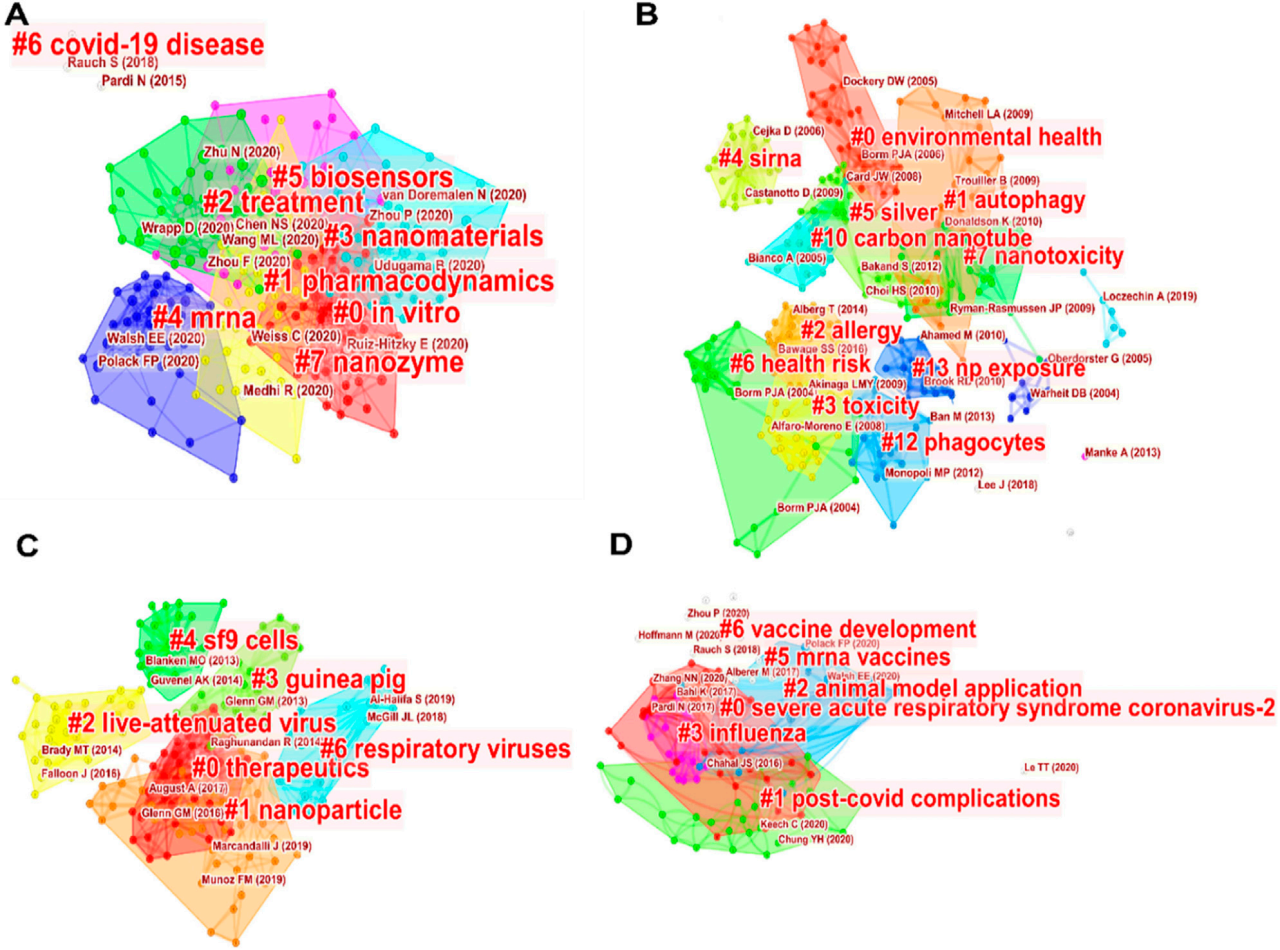
Bibliogarphic coupling analysis charts based on second-level analysis of different subareas. **(A)** COVID-19. **(B)** carbon nanotube. **(C)** RSV.**(D)** mRNA vaccine.

To further understand some prevalent subareas among the hottest keywords, COVID-19, carbon nanotube, RSV and mRNA vaccine representing four critical subareas were selected to run bibliographic coupling for the following in-depth analysis (Supplementary Table S4; Figure 4). The centrality values of each node were listed in Supplementary Tables S5, S7, S9, S11.

The statistics results of bibliographic coupling analysis are initially summarized in Supplementary Table S4. The outputs of bibliographic coupling of the relevant articles in the different areas were listed in Supplementary Tables S6, S8, S10, S12. The means of silhouette coefficients in four selected subareas range from 0.84–0.97, which are all close to the maximum value of 1, suggesting that all analyses are significant (Wilczewska et al., 2012). The proportions of the coverage articles in the clustering outputs all exceed 98% in the four subareas. However, the largest subarea COVID-19 is in fact larger than the other three altogether, its mean silhouette coefficient appears to be the smallest one. To visualize the four branch areas of the relevant research in the bibliographic coupling analyses, more comprehensive details are elaborated in the following illustration.

Diagnosis, virus detection, nanomaterials, mRNA vaccines, treatment and prevention are the core research hotspots related to the keyword COVID-19, as shown in Figure 4A, which presents the nanotechnological involvements in the COVID-19 research. As seen from the Supplementary Table S5, the latest report compared pre-COVID-19 nanomedicines with newly approved nanomedicines for COVID-19, releasing clinical trial data of unapproved nanomedicines including those for respiratory diseases, which provided a broad overview of the current clinical state of nanotechnology (Anselmo and Mitragotri, 2021). Due to the rapid speed of transmission, the rapid diagnostic tests for SARS-CoV-2 virus are urgently vital, to this demand, the gold antibody nanoparticles were designed to diagnose SARS-CoV-2 infection in high-risk patients. By using these gold antibody nanoparticles, diagnosis can be obtained without sending samples to a centralized laboratory, therefore minimizing the training requirements. Because these devices can be manufactured, stored and distributed efficiently and at low cost, they are beneficial for informing decision-making as long as positive antibody tests are detected (La Marca et al., 2020). Inhalation has been demonstrated as an efficient route for nanomedicine administration, especially when applied to respiratory diseases, for example, the inhaled delivery of silver nanomedicine inhibited respiratory bacterial and viral proliferation of *Staphylococcus aureus* SARS-CoV-2 and novel influenza A (H3N2) (Zachar, 2020). In addition, the extensive research on the infection mechanisms of SARS-CoV-2 has largely inspired the development of other vaccines, such as the mRNA vaccine designed against Middle East Respiratory Syndrome (Liu et al., 2020).

For the keyword CNT (Figure 4B; Supplementary Table S7), in fact, the main research essence behind can be concluded to be nanotoxicity, biological monitoring, and allergy. The researchers focused on the lung toxicity of carbon nanotubes mainly investigated the toxicity monitoring of carbon nanotubes and the causes of toxicity (Lam et al., 2006). In addition, the toxicological studies mainly estimate the adverse effects of carbon nanotubes on the human respiratory tract and eventually the human health (Madl and Pinkerton, 2009). The latest research uses carbon nanotubes as nanosensors to detect nitric oxide (NO) gas to monitor respiratory diseases because NO can stimulate respiratory organs, cause acute and chronic poisoning, affect and harm human health (Jeong et al., 2021) Also, carbon nanotubes could be used as a filter to remove harmful particles from the air and human respiratory tract (Issman et al., 2021). Although adverse effects of CNT on human respiratory tract were recognized, recently, studies have shown that CNT can also be employed as nanomedicine to protect the human respiratory tract (Kang et al., 2009; Park and Hwang, 2014). This inspires us carbon nanotubes can also be repurposed to detect pollutants, which helps to monitor the risks of airborne hazardous materials and prevent the development of respiratory diseases.

Respiratory Syncytial Virus (RSV) is the leading cause of lower respiratory tract infection (RTI) in young children (Wolf et al., 2006), which is also highly contagious and can be transmitted from person to person. To illustrate this subarea, we conducted a second-level analysis of RSV cluster. As shown in Figure 4C and Supplementary Table S9, keywords such as the spodoptera frugiperda (sf9) cells and guinea pigs, which are used as models to produce RSV viral particles for vaccine. To be specific, RSV fusion surface glycoprotein was modified and cloned into a baculovirus vector, followed by transduction the sf9 cells. Recombinant glycoproteins are glycosylated and cleaved into peptides in the sf9 cells. Viral nanoparticles with fusion surface glycoproteins were then extracted and purified from sf9 cells, which can be applied as the RSV vaccines (Smith et al., 2012). The production of RSV viral vaccines can be well implemented, while the issue of delivery became challenging, thus recently much research attention has been shifted in developing vaccine delivery methods, vaccine nanocarriers, etc. (Karron, 2021).

As one of the most state-of-the-art delivery approaches, the successful development of the LNP-based SARS-CoV-2 mRNA vaccines brought about a revolutionary era of mRNA therapeutics for infectious respiratory diseases. In the map of mRNA vaccine keyword shown in Figure 4D, the research focus is mainly on RSV virus, influenza virus, and the research of using LNPs for delivery. As shown in Supplementary Table S11, researchers began to focus on preparing mRNA vaccines and using nanoparticles as the carrier for vaccine delivery, aiming to improve physical stabilities of nanoparticles, the immunogenicity of mRNA vaccines as well as the preparation process (Hassett et al., 2021). Among the nanoparticles used as the mRNA delivery, LNPs is a state-of-the-art carrier, which was demonstrated to remarkably facilitate cellular uptake, enhance delivery and vaccination efficacy (Nitika and Hui, 2021). Apart from the LNPs, other nanoparticles such as cationic polymeric vectors (Yue and Wu, 2013) in formulating the mRNA vaccines are also being studied extensively as well to overcome current patent barriers of LNPs (Alabi et al., 2013). Thus, more research will be focused on fabricating more efficient nanoparticles that can improve the safety and immunogenicity of the nano-vaccines.

## 4 Discussion

To comprehensively summarize this emerging and increasingly important area, this study investigated to show the whole landscape and focus some major and urgent branch fields. However, some limitations of this study should be acknowledged. This work was based on dataset from public databases, the biases resulting from confounding factors might exist. In addition, we made conclusions based on the published articles. Such strategy has ignored those works published as patents, which may be also very important for research and development. However, WoSCC was the mostly used database for scientometrics research, and it comprised different journals all over the world, which ensures our present study rationally reflect the whole scenario of this area. Different scientometrics studies were performed based on articles on nanoparticles used in respiratory diseases from 2006 to 2021. It can be concluded that respiratory diseases and nanoparticles have become prominent research hotspots in recent years. Drug delivery and the preparation of vaccines using nanoparticles as carriers are the cutting-edge directions in this research area. Despite the adverse impacts of nanoparticles that would cause respiratory diseases, the other advantages of nanomedicine could offer potent alternatives to combat the diseases. Nano-based drugs and nucleic acids based nanovaccines have been studied and developed very rapidly and seen the promising success in recent 2 years. Overall, we believe our study has presented the constructive view in this area, allowing for referrable and predictable results for the relevant researchers. We envision that more nanotechnology will get involved in studying or treating different respiratory diseases in the ongoing period of coronavirus pandemic and even a long term post the end of this pandemic.

## Data Availability

The original contributions presented in the study are included in the article/Supplementary Material, further inquiries can be directed to the corresponding authors.
