# Supplementary material for: A scientometrics study of the nanomedicines assisted in respiratory diseases
Source: Front Bioeng Biotechnol. 2022 Dec 2;10:1053653. doi: 10.3389/fbioe.2022.1053653 (PMC9757136; doi:10.3389/fbioe.2022.1053653)
Supplement: Supplementary file 1 [file DataSheet1.docx]

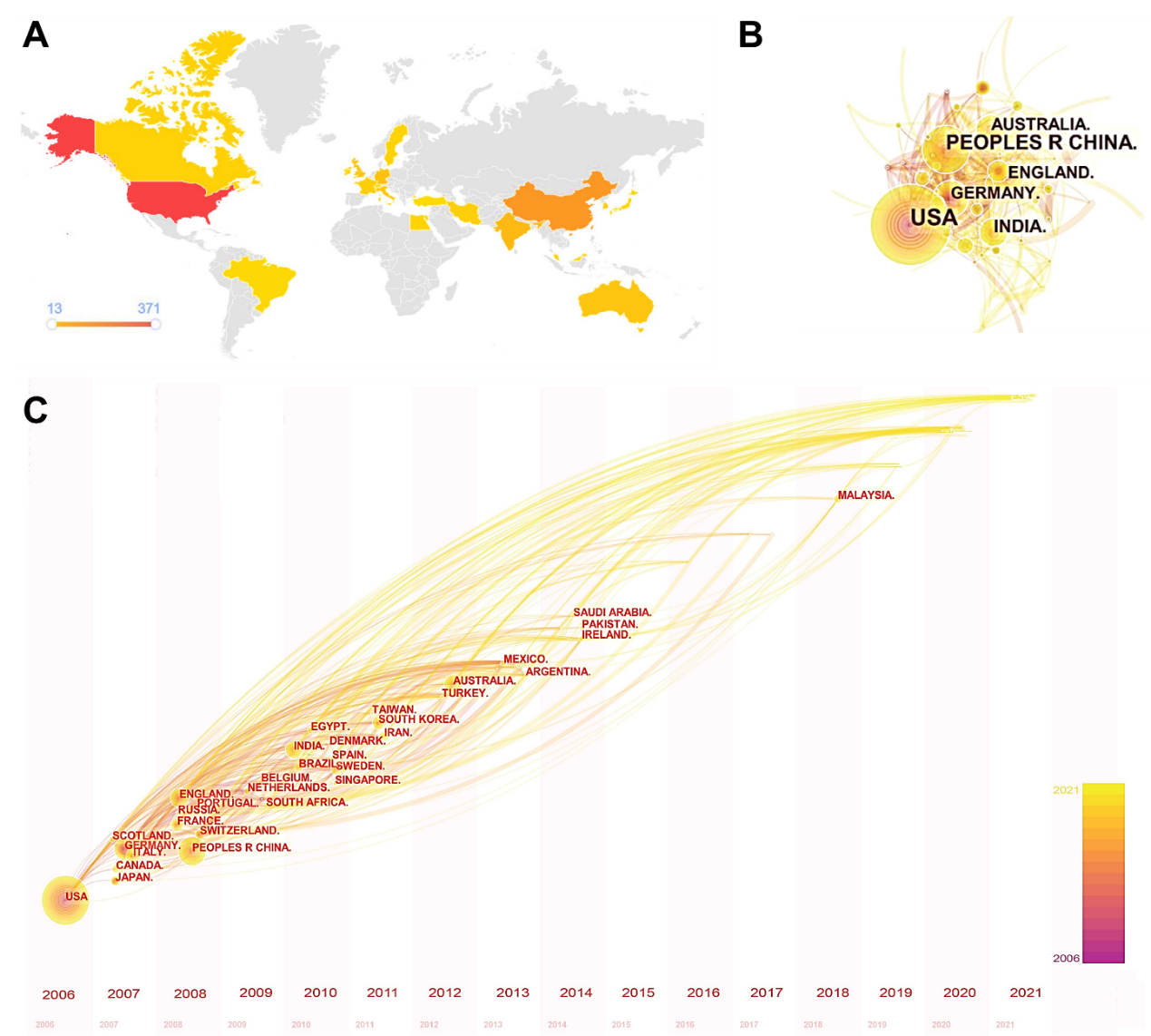
 **Supplementary Figure 1** Analysis of countries involved in the area of nanomedicines used in respiratory diseases. (A) intensity map of countries. (B) links between each country. (C) the timeline when countries started to publish in this area.


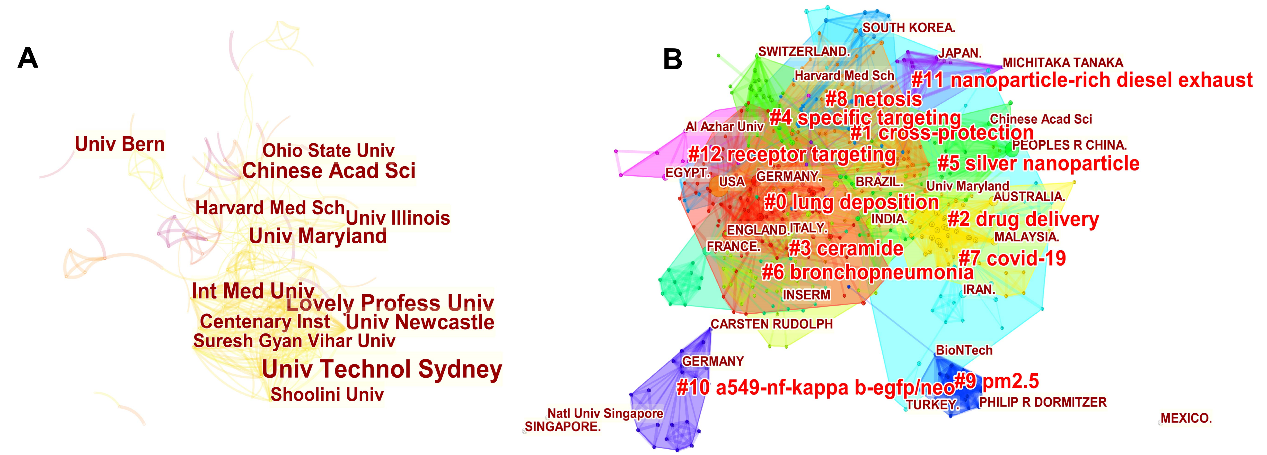


**Supplementary Figure 2** Analysis of institutions involved in the area of nanoparticles used in respiratory diseases. (A) Mapping of different prolific institutions. (B) Institutions’ locations and their main directions in this area.

**
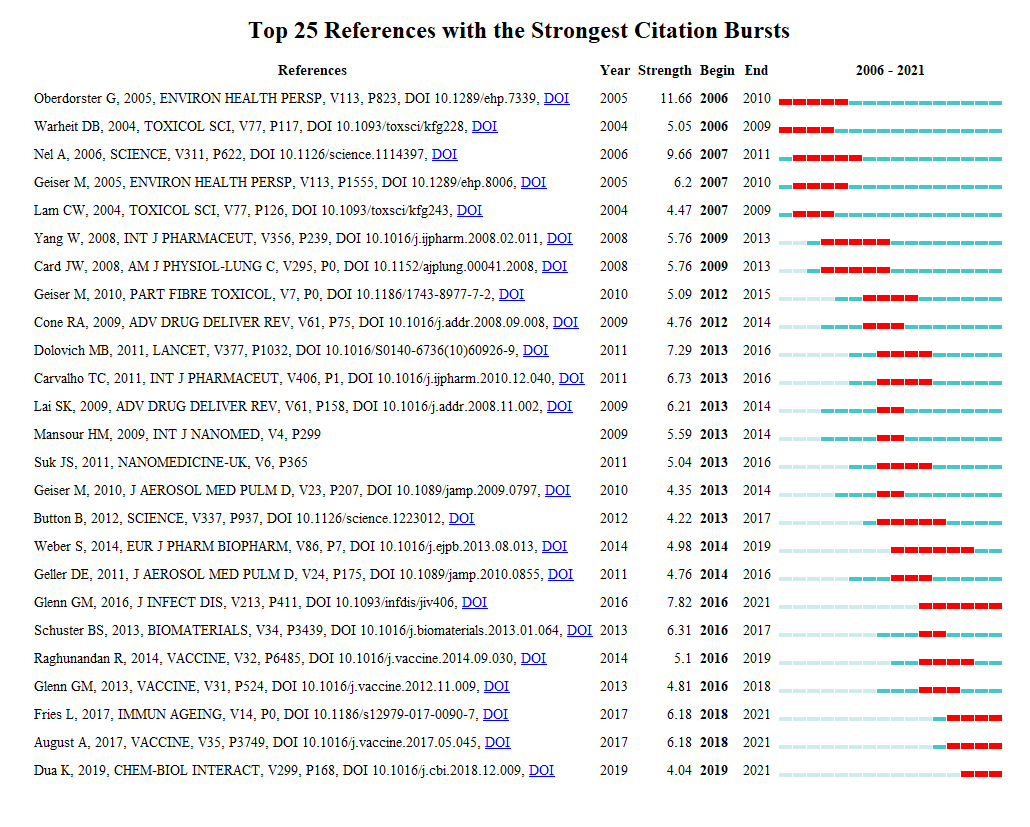
**

**Supplementary Figure 3** The top 25 references with strongest citation burst in time order.

**Supplementary Table 1** Statistical assays of the regression simulations in Figure ~~S~~1B.

| Model | R | R Square | Adjusted R Square | Std. Error  of the  Estimate | F-value |
| --- | --- | --- | --- | --- | --- |
| Linear | 0.794 | 0.631 | 0.605 | 52.612 | 23.948 |
| Exponential | 0.986 | 0.972 | 0.970 | 0.186 | 481.313 |
| Logistic  (upper bound  =1000) | 0.980 | 0.959 | 0.957 | 0.240 | 331.019 |

**Supplementary Table 2** Top 10 countries with high connectivity density.

| Country | Documents | Citations | Total link strength |
| --- | --- | --- | --- |
| USA | 371 | 21797 | 285 |
| India | 108 | 2366 | 161 |
| England | 80 | 7477 | 148 |
| Australia | 73 | 1429 | 130 |
| Germany | 86 | 9111 | 109 |
| Peoples R China | 195 | 3465 | 105 |
| Malaysia | 34 | 570 | 83 |
| Italy | 63 | 2500 | 72 |
| Canada | 41 | 2792 | 61 |
| France | 50 | 2061 | 60 |

**Supplementary Table 3** Top 15 articles with high centrality values.

| Count | Centrality | Year | Cited References |
| --- | --- | --- | --- |
| 16 | 0.40 | 2019 | AI-Halifa S, 2019, *Frontiers in immunology*, V10, P0, Nanoparticle-based vaccines against respiratory viruses. |
| 2 | 0.35 | 2012 | Garlapati S, 2012, *Vaccine*, V30, P5206, Enhanced immune responses and protection by vaccination with respiratory syncytial virus fusion protein formulated with CpG oligodeoxynucleotide and innate defense regulator peptide in polyphosphazene microparticles. |
| 4 | 0.35 | 2014 | Hwang HS, 2014, *Antiviral research*, V110, P115, Co-immunization with virus-like particle and DNA vaccines induces protection against respiratory syncytial virus infection and bronchiolitis. |
| 10 | 0.33 | 2014 | Weber S, 2014, *European Journal of Pharmaceutics and Biopharmaceutics*, V86, P7, Solid lipid nanoparticles (SLN) and nanostructured lipid carriers (NLC) for pulmonary application: a review of the state of the art. |
| 19 | 0.32 | 2016 | Glenn GM, 2016, *The Journal of infectious diseases*, V213, P411, A randomized, blinded, controlled, dose-ranging study of a respiratory syncytial virus recombinant fusion (F) nanoparticle vaccine in healthy women of childbearing age. |
| 12 | 0.30 | 2011 | Carvalho TC, 2011, *International journal of pharmaceutics*, V406, P1, Influence of particle size on regional lung deposition–what evidence is there? |
| 2 | 0.29 | 2017 | Blank F, 2017, *Journal of Nanobiotechnology*, V15, P0, Interaction of biomedical nanoparticles with the pulmonary immune system. |
| 2 | 0.29 | 2017 | Kang S, 2017, *Journal of controlled release*, V256, P56, Effects of gold nanoparticle-based vaccine size on lymph node delivery and cytotoxic T-lymphocyte responses. |
| 3 | 0.29 | 2015 | Agent P, 2015, *Breathe*, V11, P111, Inhaled therapy in cystic fibrosis: agents, devices and regimens. |
| 5 | 0.24 | 2008 | Bailey MM, 2008, *Langmuir*, V24, P13614, Pure insulin nanoparticle agglomerates for pulmonary delivery. |
| 5 | 0.22 | 2013 | Blank F, 2013, *American journal of respiratory cell and molecular biology*, V49, P67, Size-dependent uptake of particles by pulmonary antigen-presenting cell populations and trafficking to regional lymph nodes. |
| 2 | 0.21 | 2014 | Dekali S, 2014, *Toxicology reports*, V1, P157, Assessment of an in vitro model of pulmonary barrier to study the translocation of nanoparticles. |
| 2 | 0.18 | 2017 | Brockman SM, 2017, *PLoS One*, V12, P0, Dendrimer-based selective autophagy-induction rescues ΔF508-CFTR and inhibits Pseudomonas aeruginosa infection in cystic fibrosis. |
| 3 | 0.18 | 2016 | Fromen CA, 2016, *Nanomedicine: Nanotechnology, Biology and Medicine*, V12, P677, Nanoparticle surface charge impacts distribution, uptake and lymph node trafficking by pulmonary antigen-presenting cells. |
| 3 | 0.18 | 2013 | Hardy CL, 2013, *The journal of immunology*, V191, P5278, Differential uptake of nanoparticles and microparticles by pulmonary APC subsets induces discrete immunological imprints. |

**Supplementary Table 4** Bibliographic coupling analyses of the literature in four subareas*

| Subareas | Articles | Clusters（size>=2） | | Coverage articles | | Mean size | Mean silhouette | Top 3 clusters(plus ties) ordered by size (labeled by Log-likelihood ration, p-value) |
| --- | --- | --- | --- | --- | --- | --- | --- | --- |
| A: COVID-19 | 237 | | 8 | | 234 | 33 | 0.8432 | mRNA (9.87, 0.005); mRNA vaccines (9.87, 0.005); nanomaterials (9.84, 0.005) |
| B: carbon nanotube | 65 | | 11 | | 64 | 24 | 0.9714 | siRNA (5.62, 0.05); gene silencing (5.62, 0.05); viral vector (5.62, 0.05) |
| C: respiratory syncytial virus (RSV) | 59 | | 6 | | 59 | 30.33 | 0.8943 | nanoparticle (6.1, 0.05); therapeutics (4.99, 0.05); respiratory viruses (4.85, 0.05) |
| D:mRNA vaccine | 35 | | 6 | | 34 | 17.5 | 0.9214 | animal model application (3.04, 0.1); vaccine development (3.04, 0.1); severe acute respiratory syndrome coronavirus-2 (3.32, 0.1) |

* The detailed clustering results for the literature of the four subareas are shown in the appendix. (supplementary material Table S6, S8, S10 and S12).

**Supplementary Table 5** Top 10 articles with high centrality values of COVID-19 subareas.

| Count | Centrality | Year | Cite References |
| --- | --- | --- | --- |
| 15 | 0.19 | 2020 | Liu C, 2020, *ACS Central Science*, V6, P135, Research and Development on Therapeutic Agents and Vaccines for COVID-19 and Related Human Coronavirus Diseases. |
| 8 | 0.09 | 2020 | Amanat F, 2020, *Immunity*, V52, P583, SARS-CoV-2 Vaccines: Status Report. |
| 12 | 0.09 | 2019 | Kerry RG, 2019, *Nanomedicine: Nanotechnology, Biology and Medicine*, V18, P196, Nano-based approach to combat emerging viral (NIPAH virus) infection. |
| 11 | 0.08 | 2016 | Du T, 2016, *Carbon*, V110, P278, Carbon dots as inhibitors of virus by activation of type I interferon response. |
| 10 | 0.08 | 2020 | Gao JJ, 2020, *Bioscience trends*, V14, P72, Breakthrough: Chloroquine phosphate has shown apparent efficacy in treatment of COVID-19 associated pneumonia in clinical studies. |
| 34 | 0.08 | 2020 | Qiu GG, 2020, *ACS Nano*, V14, P5268, Dual-Functional Plasmonic Photothermal Biosensors for Highly Accurate Severe Acute Respiratory Syndrome Coronavirus 2 Detection. |
| 13 | 0.07 | 2020 | Bai Y, 2020, *Jama*, V323, P1406, Presumed Asymptomatic Carrier Transmission of COVID-19. |
| 9 | 0.07 | 2020 | Chen L, 2020, *Materials Science and Engineering: C*, V112, P0, An overview of functional nanoparticles as novel emerging antiviral therapeutic agents. |
| 10 | 0.07 | 2020 | Lurie N, 2020, *New England journal of medicine*, V382, P1969, Developing Covid-19 Vaccines at Pandemic Speed. |
| 13 | 0.07 | 2020 | Zhao Z, 2020, *Simple Magnetic Nano*, V0, P0, A simple magnetic nanoparticles-based viral RNA extraction method for efficient detection of SARS-CoV-2. |

**Supplementary Table 6** Bibliographic coupling analyses of the literature in COVID-19 subareas.

| Cluster ID | Size | Silhouette | Mean(Year) |
| --- | --- | --- | --- |
| 0 | 60 | 0.795 | 2018 |
| 1 | 47 | 0.804 | 2019 |
| 2 | 45 | 0.82 | 2019 |
| 3 | 44 | 0.883 | 2019 |
| 4 | 36 | 0.918 | 2019 |
| 5 | 26 | 0.858 | 2019 |
| 6 | 4 | 1 | 2018 |
| 7 | 2 | 1 | 2020 |

**Supplementary Table 7** Top 10 articles with high centrality values of Carbon nanotube subareas.

| Count | Centrality | Year | Cite References |
| --- | --- | --- | --- |
| 7 | 0.32 | 2010 | Donaldson K, 2020, *Particle and fibre toxicology*, V7, P0, Asbestos, carbon nanotubes and the pleural mesothelium: a review of the hypothesis regarding the role of long fibre retention in the parietal pleura, inflammation and mesothelioma. |
| 3 | 0.25 | 2010 | Choi HS, 2010, *Nature biotechnology*, V28, P1300, Rapid translocation of nanoparticles from the lung airspaces to the body. |
| 2 | 0.22 | 2008 | Card JW, 2008, *American Journal of Physiology-Lung Cellular and Molecular Physiology*, V295, P0, Pulmonary applications and toxicity of engineered nanoparticles. |
| 4 | 0.16 | 2006 | Borm PJA, 2006, *Particle and fibre toxicology*, V3, P0, The potential risks of nanomaterials: a review carried out for ECETOC. |
| 2 | 0.16 | 2010 | Ahamed M, 2010, *Biochemical and biophysical research communications*, V396, P578, Genotoxic potential of copper oxide nanoparticles in human lung epithelial cells. |
| 2 | 0.12 | 2013 | Ban M, 2013, *Toxicology letters*, V216, P31, Iron oxide particles modulate the ovalbumin-induced Th2 immune response in mice. |
| 2 | 0.11 | 2010 | Brook RD, 2010, *Circulation*, V121, P2231, American Heart Association Council on Epidemiology and Prevention, Council on the Kidney in Cardiovascular Disease, and Council on Nutrition, Physical Activity and Metabolism. Particulate matter air pollution and cardiovascular disease: an update to the scientific statement from the American Heart Association. |
| 2 | 0.11 | 2011 | Foldbjerg R, 2011, *Archives of toxicology*, V85, P743, Cytotoxicity and genotoxicity of silver nanoparticles in the human lung cancer cell line, A549. |
| 2 | 0.10 | 2006 | Bottini M, 2006, *Toxicology letters*, V160, P121, Multi-walled carbon nanotubes induce T lymphocyte apoptosis. |
| 2 | 0.10 | 2005 | Blanco A, 2005, *Current opinion in chemical biology*, V9, P674, Applications of carbon nanotubes in drug delivery. |

**Supplementary Table 8** Bibliographic coupling analyses of the literature in carbon nanotube subareas.

| Cluster ID | Size | Silhouette | Mean(Year) |
| --- | --- | --- | --- |
| 0 | 35 | 1 | 2015 |
| 1 | 28 | 0.941 | 2010 |
| 2 | 26 | 1 | 2014 |
| 3 | 26 | 1 | 2009 |
| 4 | 25 | 1 | 2007 |
| 5 | 25 | 0.994 | 2010 |
| 6 | 24 | 0.948 | 2004 |
| 7 | 22 | 0.925 | 2011 |
| 10 | 18 | 0.931 | 2005 |
| 12 | 18 | 0.953 | 2011 |
| 13 | 17 | 0.953 | 2009 |

**Supplementary Table 9** Top 10 articles with high centrality values of RSV subareas.

| Count | Centrality | Year | Cited References |
| --- | --- | --- | --- |
| 4 | 0.18 | 2013 | Blanken MO, 2013, *New England Journal of Medicine*, V368, P1791, Respiratory Syncytial Virus and Recurrent Wheeze in Healthy Preterm Infants. |
| 14 | 0.08 | 2017 | August A, 2017, *Vaccine*, V35, P3749, A Phase 2 randomized, observer-blind, placebo-controlled, dose-ranging trial of aluminum-adjuvanted respiratory syncytial virus F particle vaccine formulations in healthy women of childbearing age. |
| 9 | 0.07 | 2013 | Glenn GM, 2013, *Vaccine*, V31, P524, Safety and immunogenicity of a Sf9 insect cell-derived respiratory syncytial virus fusion protein nanoparticle vaccine. |
| 3 | 0.07 | 2012 | Bernstein DI, 2012, *The Pediatric infectious disease journal*, V31, P109, Phase 1 Study of the Safety and Immunogenicity of a Live, Attenuated Respiratory Syncytial Virus and Parainfluenza Virus Type 3 Vaccine in Seronegative Children. |
| 14 | 0.06 | 2017 | Fries L, 2017, *Immunity & Ageing*, V14, P0, Immunogenicity and safety of a respiratory syncytial virus fusion protein (RSV F) nanoparticle vaccine in older adults. |
| 11 | 0.06 | 2017 | Shi T, 2017, *Lancet*, V390, P946, Global, regional, and national disease burden estimates of acute lower respiratory infections due to respiratory syncytial virus in young children in 2015: a systematic review and modelling study. |
| 8 | 0.05 | 2015 | Habibi MS, 2015, *American journal of respiratory and critical care medicine*, V191, P1040, Impaired Antibody-mediated Protection and Defective IgA B-Cell Memory in Experimental Infection of Adults with Respiratory Syncytial Virus. |
| 4 | 0.05 | 2014 | Brady MT, 2014, *Pediatrics*, V134, P415, Updated Guidance for Palivizumab Prophylaxis Among Infants and Young Children at Increased Risk of Hospitalization for Respiratory Syncytial Virus Infection. |
| 4 | 0.05 | 2018 | Beran J, 2018, *The Journal of infectious diseases*, V217, P1616, Safety and Immunogenicity of 3 Formulations of an Investigational Respiratory Syncytial Virus Vaccine in Nonpregnant Women: Results From 2 Phase 2 Trials. |
| 2 | 0.05 | 2019 | Alansari K, 2019, *Pediatrics*, V143, P0, Monoclonal Antibody Treatment of RSV Bronchiolitis in Young Infants: A Randomized Trial. |

**Supplementary Table 10** Bibliographic coupling analyses of the literature in respiratory syncytial virus (RSV) subareas.

| Cluster ID | Size | Silhouette | Mean(Year) |
| --- | --- | --- | --- |
| 0 | 45 | 0.814 | 2016 |
| 1 | 41 | 0.858 | 2017 |
| 2 | 31 | 0.938 | 2013 |
| 3 | 24 | 0.9 | 2014 |
| 4 | 22 | 0.983 | 2010 |
| 6 | 19 | 0.981 | 2018 |

**Supplementary Table 11** Top 10 articles with high centrality values of mRNA vaccine subareas.

| Count | Centrality | Year | Cited References |
| --- | --- | --- | --- |
| 8 | 0.28 | 2020 | Mulligan MJ, 2020, *Nature*, V586, P589, Phase I/II study of COVID-19 RNA vaccine BNT162b1 in adults. |
| 3 | 0.22 | 2016 | Reichmuth AM, 2016, *Therapeutic delivery*, V7, P319, mRNA vaccine delivery using lipid nanoparticles. |
| 7 | 0.18 | 2019 | Feldman RA, 2019, *Vaccine*, V37, P3326, mRNA vaccines against H10N8 and H7N9 influenza viruses of pandemic potential are immunogenic and well tolerated in healthy adults in phase 1 randomized clinical trials. |
| 3 | 0.17 | 2020 | Gao Q, 2020, *Science*, V369, P77, Development of an inactivated vaccine candidate for SARS-CoV-2. |
| 6 | 0.15 | 2017 | Bahl K, 2017, *Molecular Therapy*, V25, P1316, Preclinical and clinical demonstration of immunogenicity by mRNA vaccines against H10N8 and H7N9 influenza viruses. |
| 4 | 0.15 | 2020 | Mercado NB, 2020, *Nature*, V586, P583, Single-shot Ad26 vaccine protects against SARS-CoV-2 in rhesus macaques. |
| 4 | 0.15 | 2016 | Brazzoli M, 2016, *Journal of virology*, V90, P332, Induction of broad-based immunity and protective efficacy by self-amplifying mRNA vaccines encoding influenza virus hemagglutinin. |
| 6 | 0.14 | 2020 | McKay PF, 2020, *Nature communications*, V11, P0, Self-amplifying RNA SARS-CoV-2 lipid nanoparticle vaccine candidate induces high neutralizing antibody titers in mice. |
| 8 | 0.12 | 2020 | Sahin U, 2020, *Nature*, V586, P594, COVID-19 vaccine BNT162b1 elicits human antibody and TH1 T cell responses. |
| 6 | 0.12 | 2018 | Pardi N, 2018, *Nature communications*, V11, P0, Nucleoside-modified mRNA immunization elicits influenza virus hemagglutinin stalk-specific antibodies. |

**Supplementary Table 12** Bibliographic coupling analyses of the literature in mRNA vaccine subareas.

| Cluster ID | Size | Silhouette | Mean(Year) |
| --- | --- | --- | --- |
| 0 | 31 | 0.856 | 2018 |
| 1 | 23 | 0.966 | 2019 |
| 2 | 21 | 0.909 | 2019 |
| 3 | 15 | 0.956 | 2017 |
| 5 | 8 | 0.967 | 2019 |
| 6 | 7 | 0.976 | 2019 |

**Large versions of CiteSpace analysis the corresponding graphs in the main manuscript.**

**
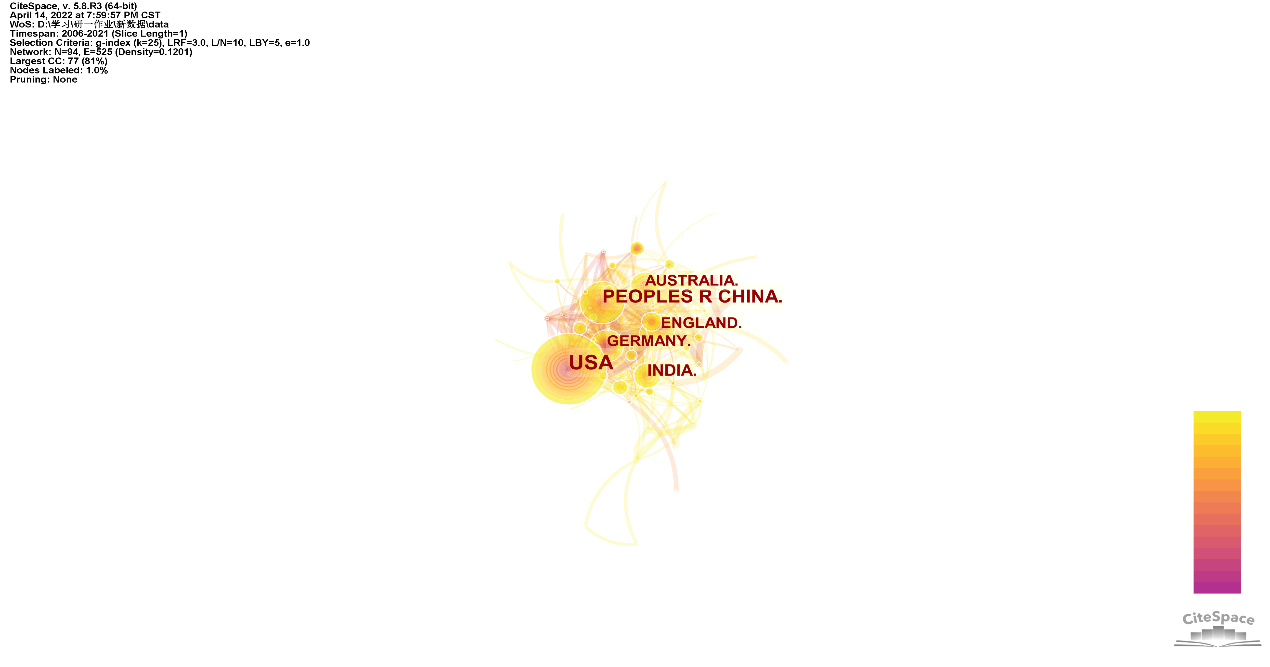
Figure S1B** Links between each country.


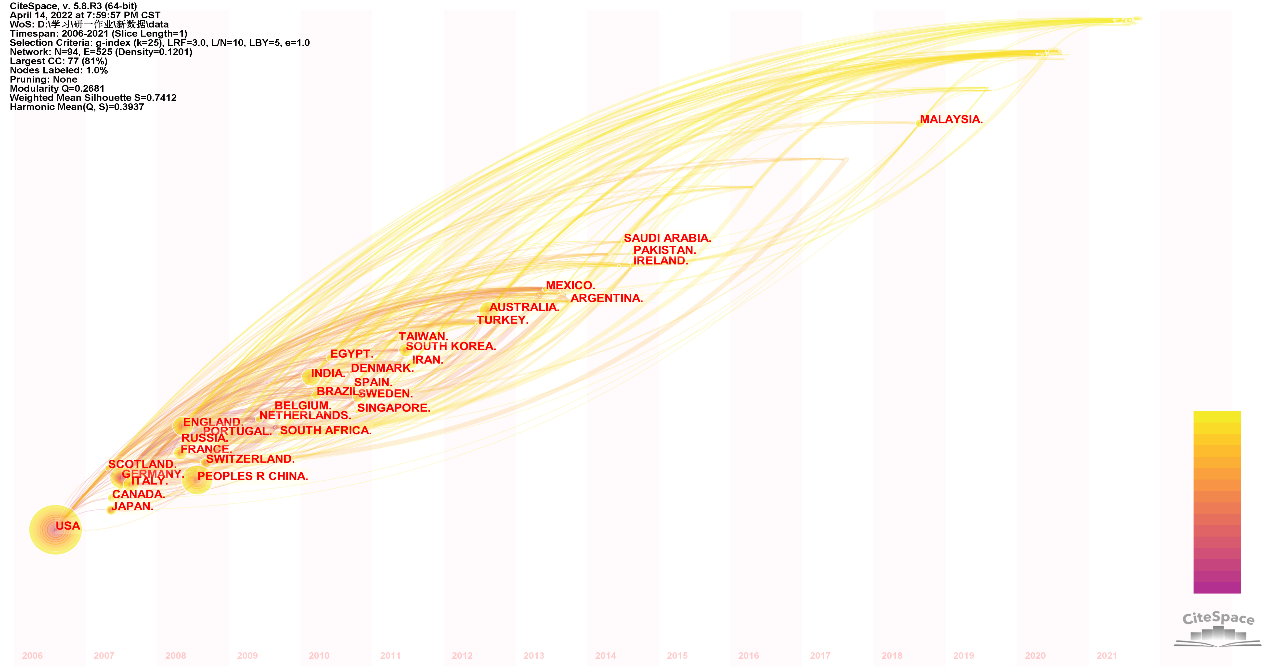


**Figure S1C** The timeline when countries started to publish in this area.


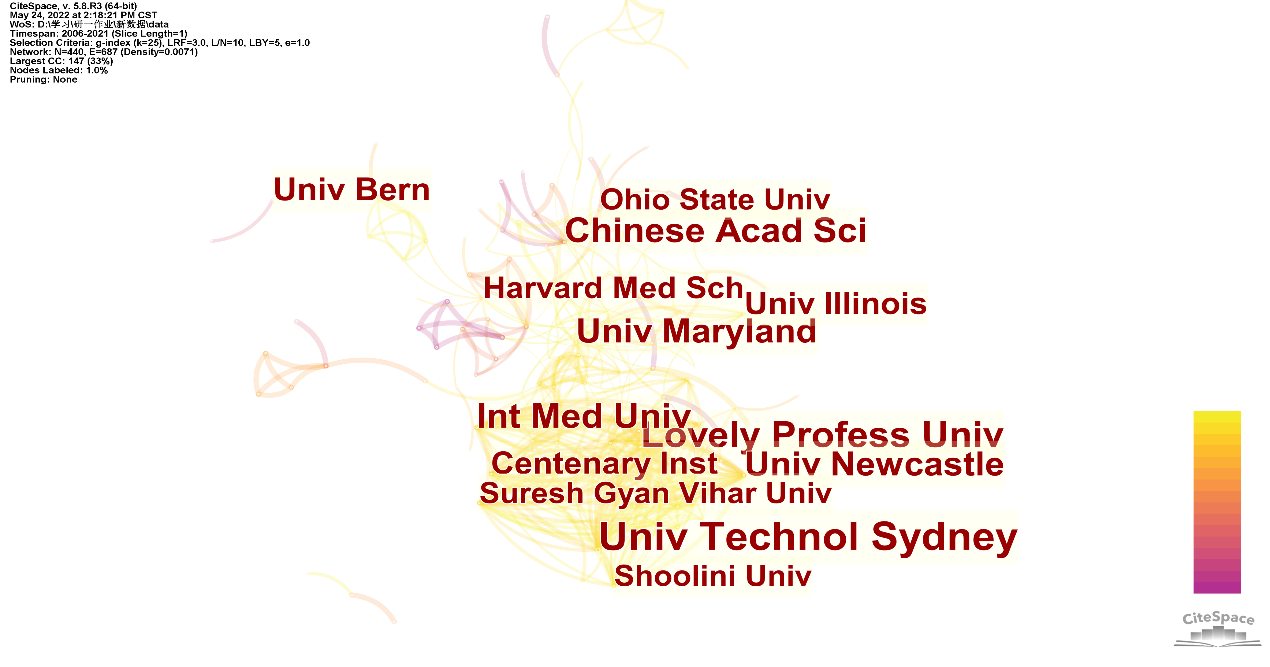


**Figure S2A** Mapping of different prolific institutions.


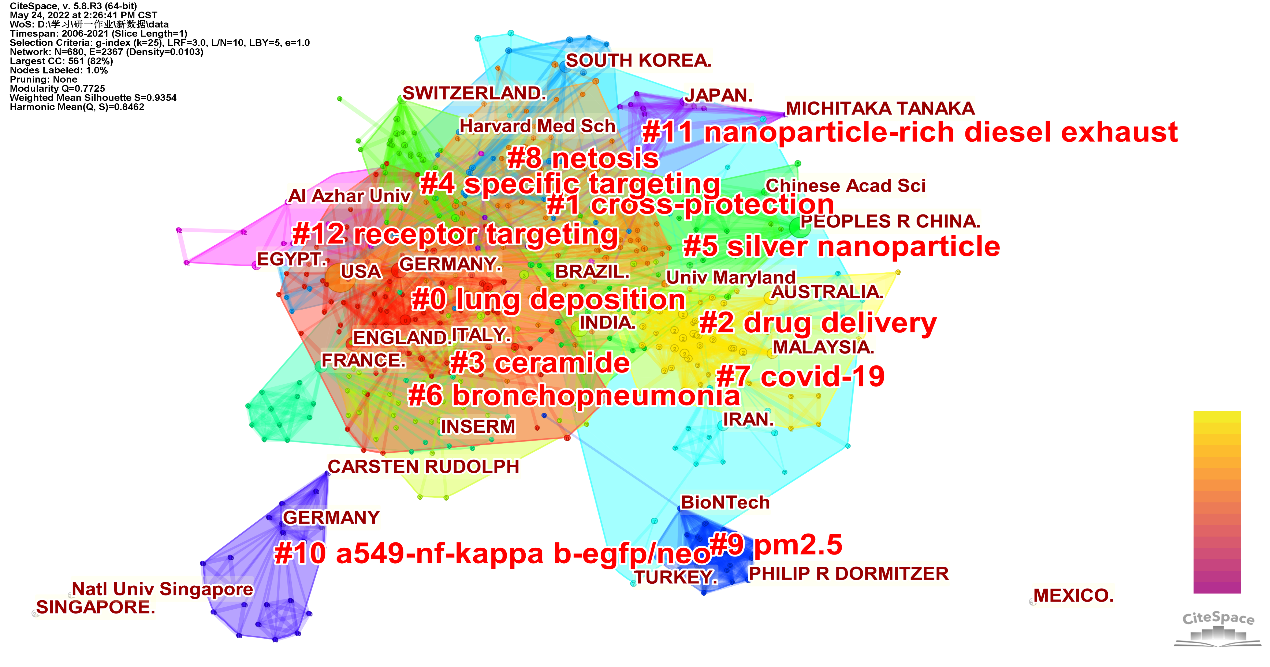


**Figure S2B** Institutions’ locations and their main directions in this area.


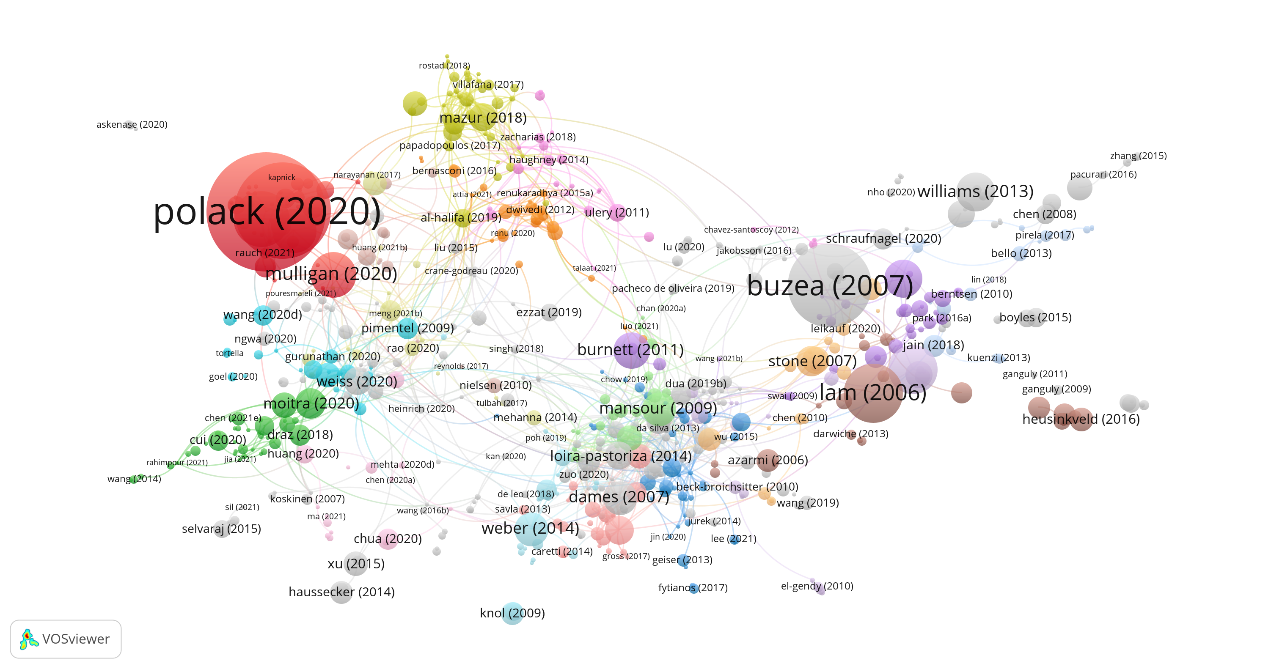
**Figure 3A** Visualization graph of cited references.


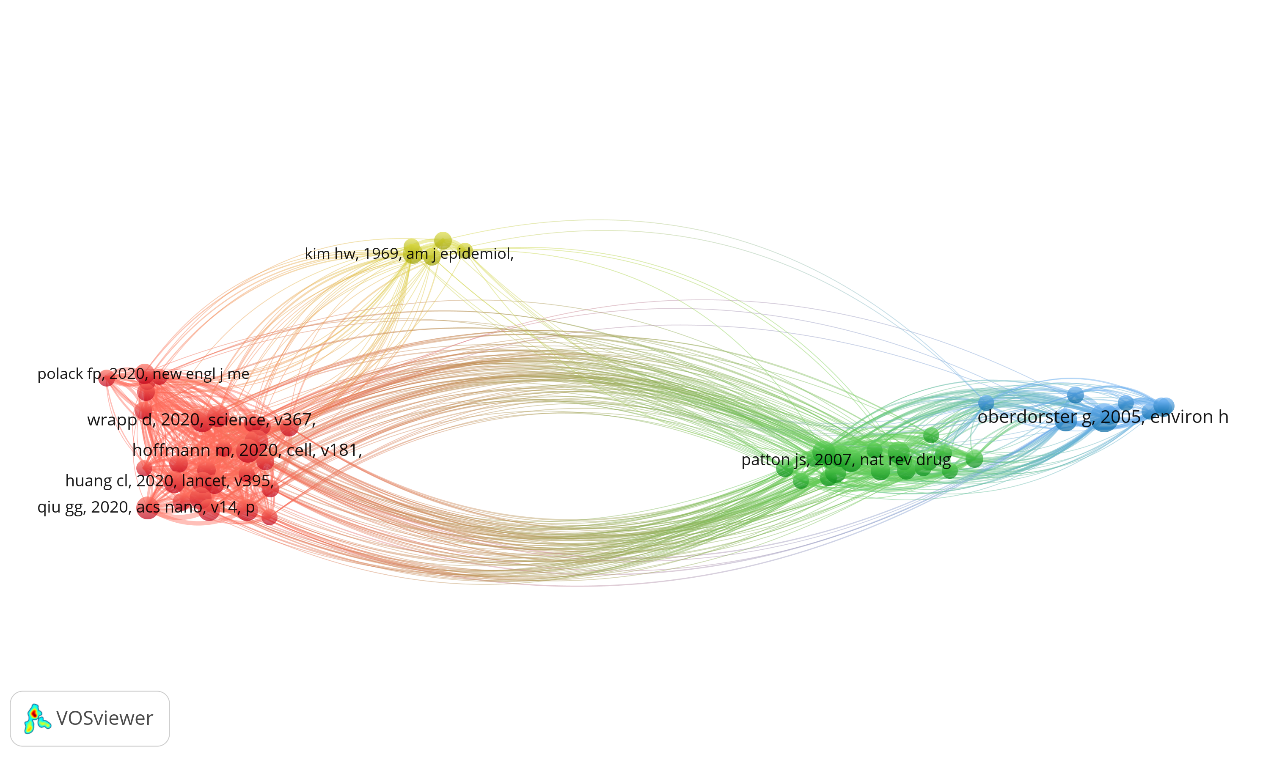
**Figure 3B** The connection between references.


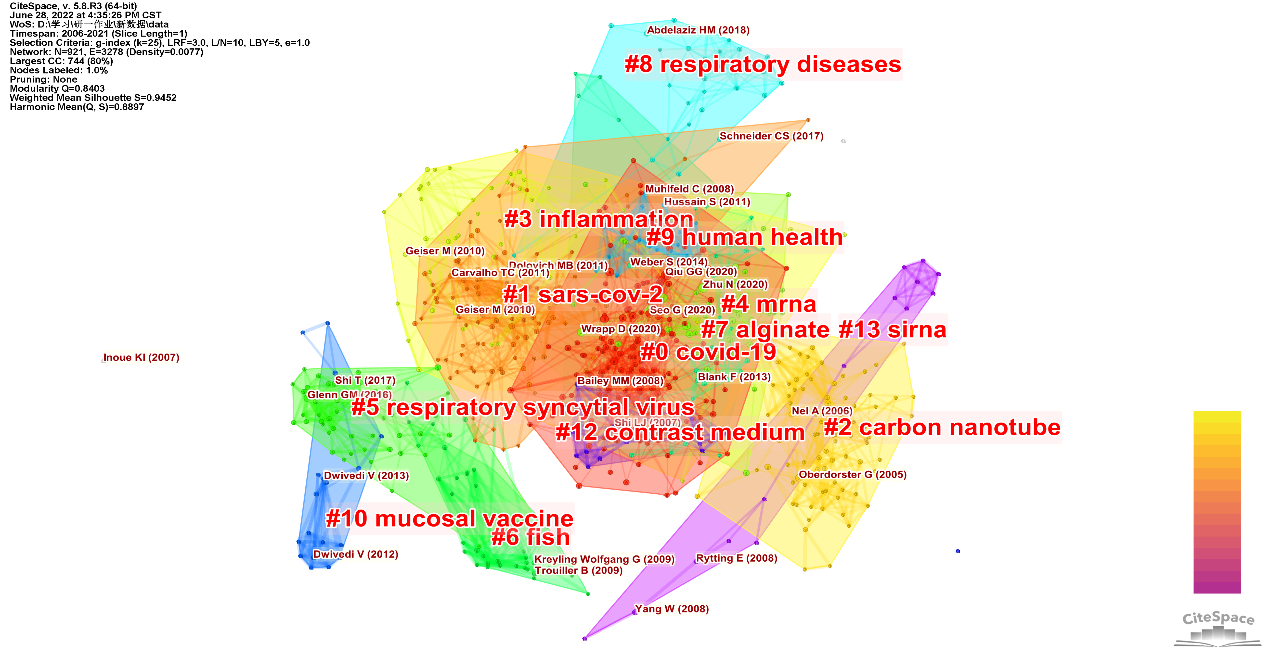


**Figure 3C** Co-citation references keywords.


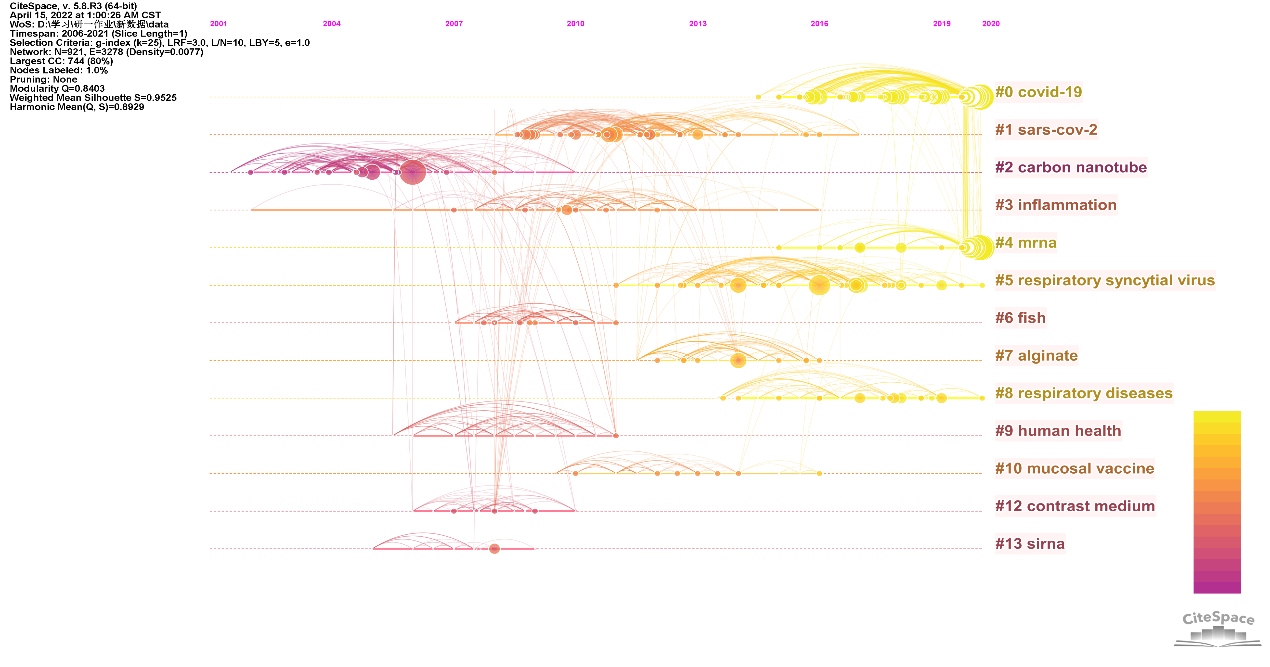


**Figure 3D** The timeline of cited references.


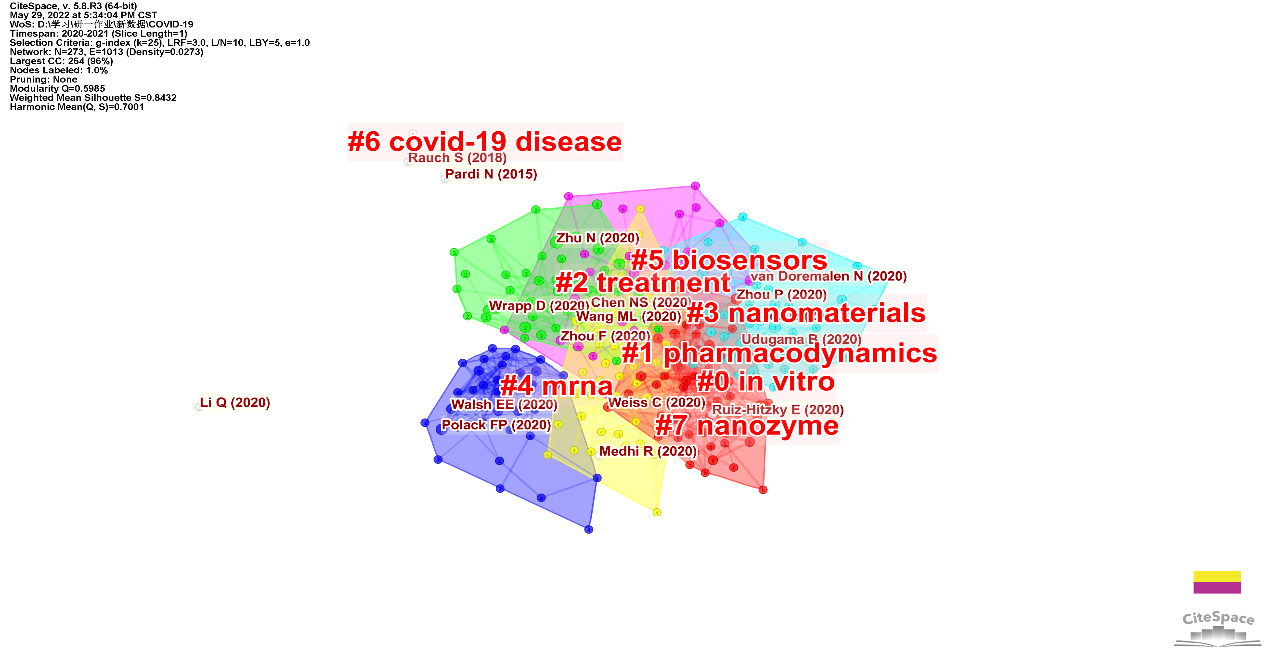


**Figure 4A** Bibliogarphic coupling analysis charts based on second-level analysis of COVID-19.


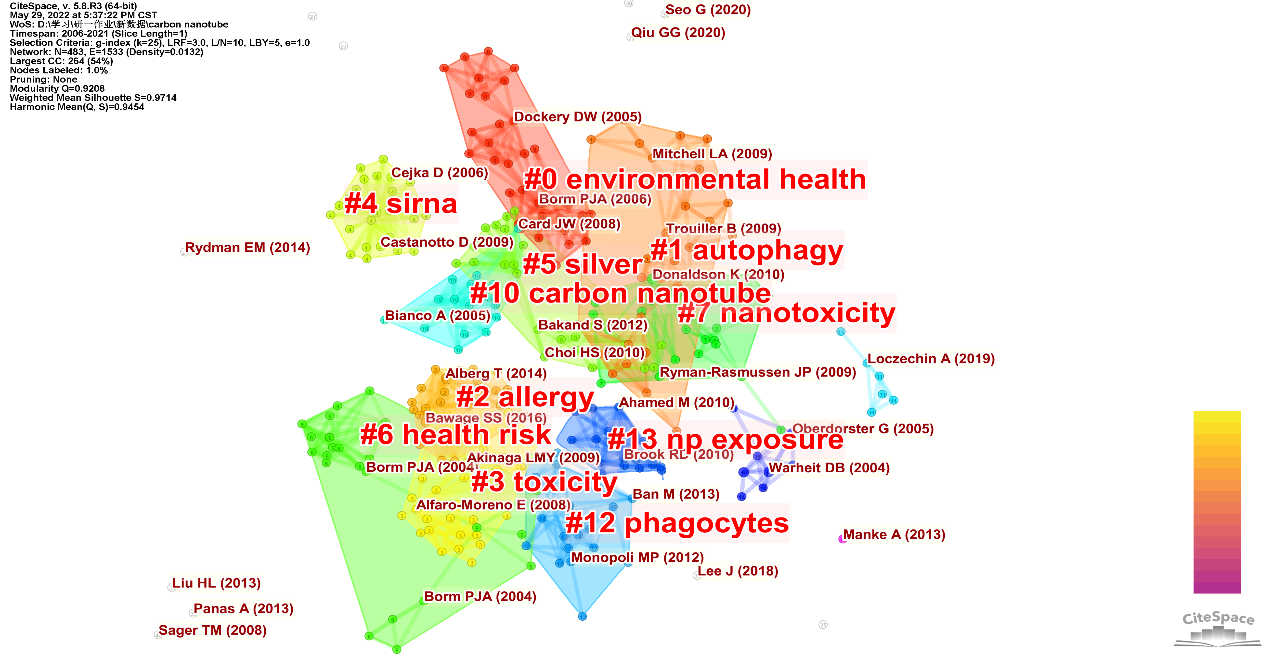


**Figure 4B** Bibliogarphic coupling analysis charts based on second-level analysis of carbon nanotube.


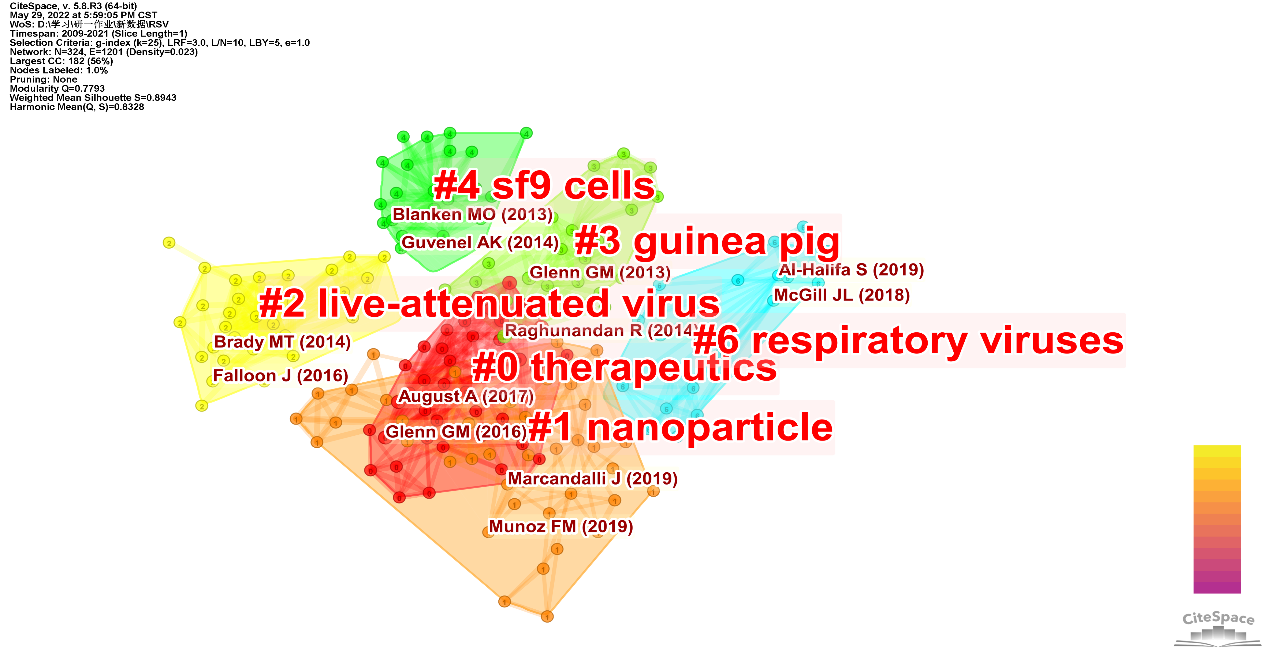


**Figure 4C** Bibliogarphic coupling analysis charts based on second-level analysis of RSV.


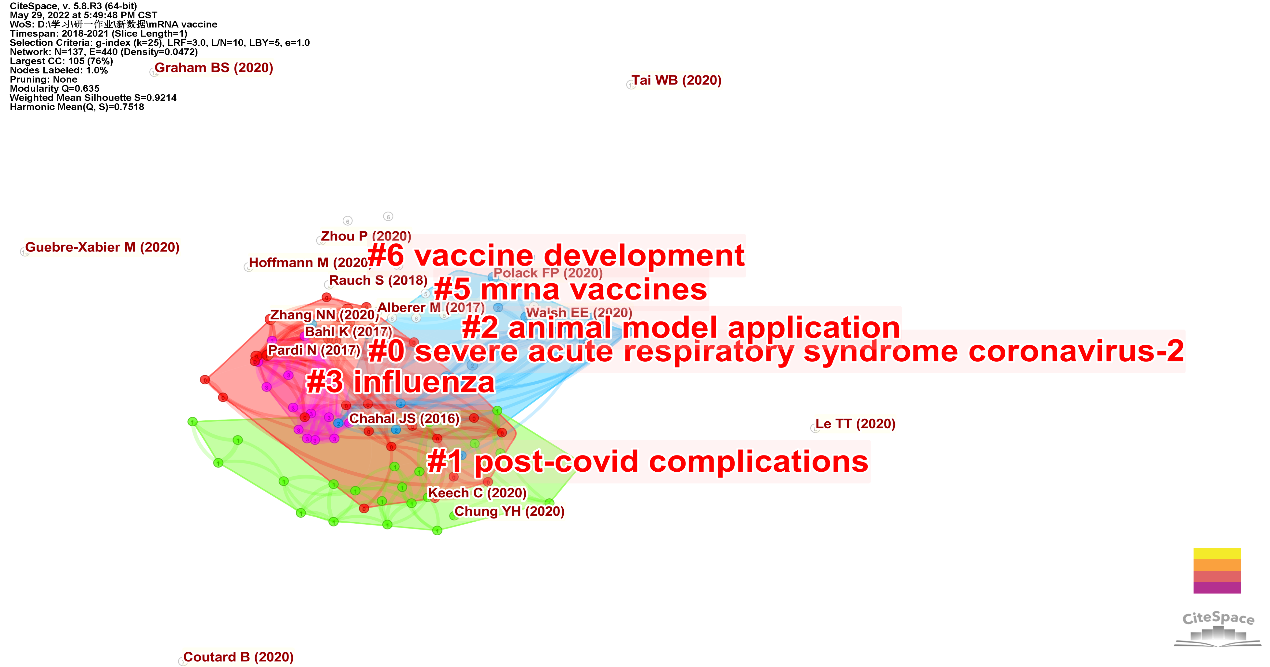


**Figure 4D** Bibliogarphic coupling analysis charts based on second-level analysis of mRNA vaccine.
